# Lower Urinary Tract Symptoms Correlate with Erectile Dysfunction and Premature Ejaculation but Not with Men’s Sexual Activity: Results from a Large Population-Representative Study

**DOI:** 10.3390/healthcare12141408

**Published:** 2024-07-15

**Authors:** Mikolaj Przydacz, Pawel Rajwa, Sabrina De Cillis, Cyrille Guillot-Tantay, Francois Herve, Antonio Tienza Fernandez, Manuela Tutolo, Mehmet Gokhan Culha, Paolo Geretto, Chen Shenhar, Marcin Miszczyk, Piotr Chlosta, Veronique Phe, Nadir Osman

**Affiliations:** 1 Department of Urology, Jagiellonian University Medical College, 31-008 Krakow, Poland; 2 Department of Urology, Medical University of Silesia, 41-808 Zabrze, Poland; 3 Department of Urology, Medical University of Vienna, 1090 Vienna, Austria; 4 Division of Urology, Department of Oncology, San Luigi Gonzaga Hospital, University of Turin, 10124 Orbassano, Italy; 5 Service d’urologie, Hôpital Foch, 92151 Suresnes, France; 6 Department of Urology, ERN Accredited Centrum, Ghent University Hospital, 9000 Ghent, Belgium; 7 Department of Urology, Son Espases University Hospital, Health Research Institute of the Balearic Islands, 07120 Palma, Spain; atienza@alumni.unav.es; 8 Unit of Urology, Division of Oncology, Urological Research Institute, IRCCS San Raffaele Hospital, 20132 Milan, Italy; 9 Department of Urology, Prof. Dr. Cemil Tascioglu City Hospital, University of Health Sciences, 34668 Istanbul, Turkey; 10 Division of Neuro-Urology, Department of Surgical Sciences, CTO Hospital, Citta della Salute e della Scienza, 10126 Turin, Italy; 11 Glickman Urological and Kidney Institute, Cleveland Clinic, Cleveland, OH 44195, USA; 12 Collegium Medicum Faculty of Medicine, WSB University, 41-300 Dabrowa Gornicza, Poland; 13 Department of Urology, Assistance Publique-Hôpitaux de Paris, Tenon Academic Hospital, Sorbonne University, 75020 Paris, France; 14 Department of Urology, Royal Hallamshire Hospital, Sheffield S10 2JF, UK

**Keywords:** lower urinary tract symptoms, sexual activity, erectile dysfunction, premature ejaculation

## Abstract

Background: Lower urinary tract symptoms (LUTS) contribute to erectile dysfunction (ED) and premature ejaculation (PE). However, only a few studies have been conducted with representative groups of men that had well-balanced demographic characteristics. Thus, we aimed to confirm the effect of LUTS on ED and PE and to analyze the association between LUTS and men’s sexual activity in a large representative cohort. In addition, we evaluated the sex-specific and overall quality of life of men who had LUTS with either ED or PE. Methods: We used the latest census and estimated the sample size to build a group of men representative of the population. LUTS, ED, and PE were evaluated with reliable instruments. Regression models were used to analyze the data. Results: All included men were representative in relation to their age and residential location (*n* = 3001). ED and PE were more common in men who reported LUTS compared with men who did not have LUTS (*p* < 0.001). Age, comorbidity, and lifestyle did not affect the negative effect of LUTS on ED or PE (regression coefficients of 0.159 and 0.528 for ED and PE, respectively, *p* < 0.001). However, regression models did not validate the impact of LUTS on sexual activity, defined by intercourse frequency and number of sexual partners (odds ratio of 0.981, CI 0.961–1.001, *p* = 0.061). Nevertheless, men with LUTS and either ED or PE had worse quality of sexual life and general quality of life compared with the remaining respondents. Conclusion: LUTS worsened ED and PE but had no impact on men’s sexual activity. Our findings confirm the recommendations to assess for LUTS in men reporting ED or PE. Clinical Trial Registration: The study was registered with ClinicalTrials.gov (NCT05462171).

## 1. Introduction

Lower urinary tract symptoms (LUTS) refer to a group of symptoms that are not disease- or condition-specific. In men, LUTS are commonly associated with bladder outlet obstruction; however, LUTS may be related to other lower urinary tract functional and anatomical abnormalities whose prevalence increases with age. Although LUTS are not considered life-threatening, they negatively affect physical and mental health, work productivity, sleep, and health-related quality of life [1].

Many investigators report that LUTS are often associated with men’s sexual dysfunctions. The European Association of Urology (EAU) Guidelines on Sexual and Reproductive Health indicate that odds ratios (ORs) of erectile dysfunction (ED) among men with LUTS range from 1.52 to 28.7, with prevalence ranging from 58% to 80% [2]. Although the association of LUTS with premature ejaculation (PE) has received less scrutiny compared with LUTS and ED, Sihotang et al., in their recent systematic review with a meta-analysis, hypothesized that there is a correlation between LUTS and PE [3]. ED and PE are the most prevalent and the most stressful of men’s sexual dysfunctions [2,4]; thus, investigators and clinicians should accurately assess LUTS/ED/PE correlations because these conditions independently impair men’s quality of life and psychological well-being [5]. However, the current literature lacks high-quality research based on large representative pools of men with well-balanced demographic characteristics and instruments that are widely accepted and professionally validated for LUTS, ED, and PE assessments [3]. Although there are some population- and community-level investigations on LUTS/ED/PE, these studies were burdened by poor objectivity in the selection of participant characteristics. For example, some studies involved patients seeking treatment at hospitals and outpatient clinics for various medical conditions [6] or respondents who belonged to exclusive patient cohorts [7,8] and specific communities [9,10]. In addition, earlier studies were performed with non-validated instruments, e.g., studies based on only a single-item, self-reported question to assess PE [11,12]. Rigorous population-representative studies provide reliable estimates and precisely uncover significant correlations that may increase awareness to promote health and highlight the potential burden to society.

Considering the aforesaid issues, we aimed to evaluate the effect of LUTS on ED and PE and the association between LUTS and men’s sexual activity in a large population-representative group of men who completed reliable LUTS, ED, and PE questionnaires. In addition, we analyzed the quality of life, both sexual and general, of men who had ED with LUTS or PE with LUTS.

## 2. Methods

This study is an extension of the ED POLAND, a representative, population-based, cross-sectional survey of Polish men’s sexual health. The extension was devised by the EAU Young Academic Urologists Working Group on Functional Urology. All objectives of this extension were prespecified in the statistical analysis plan prior to conducting the survey. We adhered to prescribed recommendations and standards for conducting observational studies [13].

### 2.1. Overview

Because of the personal nature of the topic, we surveyed participants through computer-based web interviews. Notably, our population-based analyses with web-based solutions are valid because 93.3% of Polish households had Internet access in 2022, and there was no significant difference between urban and rural populations (data from Eurostat and the Central Statistical Office of Poland [14]). To construct a sample representative of the entire Polish population, we utilized data from the latest Polish population census (2021) and incorporated age and place of residence as quota controls [15]. We included men aged 18 years or older and living in all geographical regions of Poland (i.e., in all 16 states/voivodships with adequate proportions of respondents from urban and rural areas). All data were collected by IPSOS, a certified research agency with relevant expertise (OFBOR, ESOMAR, PKJBI, and PKJPA). The data collection process also included pilot web surveys with cognitive debriefing interviews (*n* = 200), regular stratification and quality-control checks, and calculations of post-stratification weights.

### 2.2. Measures

General demographic data were collected for all respondents.

Erectile dysfunction was assessed with the five-item International Index of Erectile Function (IIEF-5), the most used and reliable questionnaire for ED evaluation [2,16]. An IIEF-5 score of 16 or less was a referent for clinically significant ED, with additional analyses of standardized levels of ED severity based on the IIEF-5 score: 22–25, without ED; 17–21, mild ED; 12–16, mild to moderate ED; 8–11, moderate ED; and 5–7, severe ED (i.e., the lower the score, the greater the severity of symptoms).

Premature ejaculation was evaluated with the Premature Ejaculation Diagnostic Tool (PEDT), a valid measuring questionnaire that embraces the multidimensional nature of PE [2,17]. A PEDT score of 11 or more was a reference for clinically significant PE, with additional standardized scale cut-off points: ≤8, without PE; 9–10, probable presence of PE; and ≥11, presence of PE (i.e., the higher the score, the greater the severity of symptoms).

Lower urinary tract symptoms were investigated with the International Prostate Symptom Score (IPSS), the most widely used instrument that evaluates the severity of LUTS in men [18]. An IPSS value was further assigned to the specific level of standardized LUTS severity: 0, no symptoms; 1–7, mild symptoms; 8–19, moderate symptoms; and 20–35, severe symptoms (i.e., the greater the score, the greater the severity of symptoms).

These three surveys underwent thorough translation, adaptation, and validation into Polish [19,20,21], and they are addressed in the pertinent EAU guidelines [2,18].

To evaluate sexual activity, we inquired about the frequency of sexual intercourse and the number of sexual partners. As a guide for these questions, we used the US General Social Survey, a nationally representative review [22].

Lastly, we included questions on sexual life quality (“In the past 4 weeks, how were you satisfied with your sex life?”), on general quality of life (“If you were to spend the rest of your life in your current condition, how would you describe your overall well-being?”), and on relevant comorbidities and lifestyle habits.

### 2.3. Statistics

Quantitative (ordinal) variables were described using measures such as means, standard deviations, medians, minimum and maximum values (ranges), and a 95% confidence interval (CI). Qualitative (categorical) variables were presented as counts and percentages. For quantitative variables, nonparametric tests were used, such as Mann–Whitney or Kruskal–Wallis with Dunn’s post hoc test, if applicable. The chi-square test or Fisher’s exact test, if the expected counts were low, were used for qualitative variables. The sample size was determined using the age distribution of the population, as obtained from the most recent census [15], with the methodology utilized in other population-based studies focusing on men’s sexual health [23]. Briefly, we estimated the sample size based on the standard deviation in the population, the expected effect size and the drop-out rate, the acceptable level of significance and margins of error, the power of the study, and the hypothesized rate of the events in the population. In addition, we analyzed general recommendations from the recent Polish census for future large population-level studies. Lastly, the sample size was set to 3000 respondents, a size that afforded a 95% certainty that the survey results would be ±1–2% of the results had we surveyed the entire adult Polish population of men.

We employed linear regression models to assess the effect of LUTS on ED and PE, presenting the outcomes as regression coefficients along with a 95% CI. Logistic regression models were utilized to analyze the effect of LUTS on sexual activity, with results reported as ORs and a 95% CI. The dependent variable was ‘no sexual activity’ (i.e., no sexual intercourse in the prior 12 months). For both regression models, questionnaire results were included as raw data (raw scores).

A statistical significance level was set at *p* < 0.05. Data analysis was performed using R (version 4.3.0, The R Project for Statistical Computing, Vienna, Austria).

## 3. Results

This analysis was based on data for 3001 men, ensuring representation across different age groups and residential areas (response rate: 51.7%).

### 3.1. Effects of LUTS on ED and PE

We found a significant correlation between LUTS and ED. Erectile dysfunction was less common in men without LUTS compared with respondents who had LUTS (Table 1, Spearman’s rank correlation coefficient R = −0.34, *p* < 0.001). The different cut-offs of IIEF-5 confirmed the associations between LUTS and ED (Table 1, *p* < 0.001).

Analogously, LUTS were significantly associated with PE. Premature ejaculation was less common in men without LUTS compared with men who reported LUTS (Table 1, Spearman’s rank correlation coefficient R = 0.34, *p* < 0.001). By employing various thresholds for PEDT, we substantiated the significant relation between LUTS and PE (Table 1, *p* < 0.001).

### 3.2. Age-Specific Effects of LUTS on ED and PE

With univariate linear regression, we analyzed the effects of LUTS on ED and PE in all age subgroups. For all age subgroups considered together, we found a significant association between LUTS and ED, with a regression coefficient of −0.224, i.e., a 1 point increase in the IPSS was reflected in an average 0.224 points decrease in the IIEF-5 score (Table 2). For each age subgroup, the regression coefficient was also statistically significant (Table 2).

We found the same relation between LUTS and PE and calculated a regression coefficient of 0.592 when considering all age subgroups together, i.e., an increase in the IPSS score by 1 point corresponded to an average increase in the PEDT score of 0.592 points (Table 2). The regression coefficient for each specific age subgroup was also statistically significant (Table 2).

### 3.3. Age-, Comorbidity-, and Lifestyle-Adjusted Effects of LUTS on ED and PE

With multivariate linear regression, we observed statistically significant effects of LUTS on ED and PE after adjusting for age, comorbidities, and lifestyle habits (Table 3). For ED, the regression coefficient was −0.159, indicating that a 1 point increase in the IPSS resulted in an average decrease of 0.159 points in the IIEF-5 score. For PE, the regression coefficient was 0.528, meaning that a 1 point increase in the IPSS led to an average increase of 0.528 points in the PEDT score.

### 3.4. Item-Specific Effects of LUTS on ED and PE

With additional multivariate linear regression, we investigated which items of the IPSS had effects on the IIEF-5 and PEDT scores (Table 4). The IIEF-5 score was negatively affected by feeling of incomplete emptying (item 1), intermittent stream (item 3), urgency (item 4), straining (item 5), and weak stream (item 6); frequency (item 2) and nocturia (item 7) did not affect the IIEF-5 scores. Items 1, 6, and 4 had the strongest effects on the IIEF-5 score, i.e., the highest absolute regression coefficients.

The IPSS items 1 (feeling of incomplete emptying), 2 (frequency), 4 (urgency), and 5 (straining) had negative effects on the PEDT score, whereas items 3, 6, and 7 did not have any effects. The strongest effects on the PEDT score were observed with items 1, 5, and 4, defined as the highest absolute regression coefficient values.

### 3.5. Effect of LUTS on Sexual Activity

Lower urinary tract symptoms significantly correlated with the frequency of sexual intercourse. Men with LUTS were characterized by a significantly lower frequency of sexual intercourse compared with respondents who did not report LUTS (*p* = 0.001). On the contrary, we did not observe a correlation between LUTS and the number of sex partners (*p* = 0.544). Moreover, multivariate logistic regression failed to prove an effect of LUTS on men’s sexual activity (OR = 0.981, CI 0.961–1.001, *p* = 0.061).

### 3.6. Effects on the Quality of Sexual Life and on General Quality of Life

**Figure 1:**
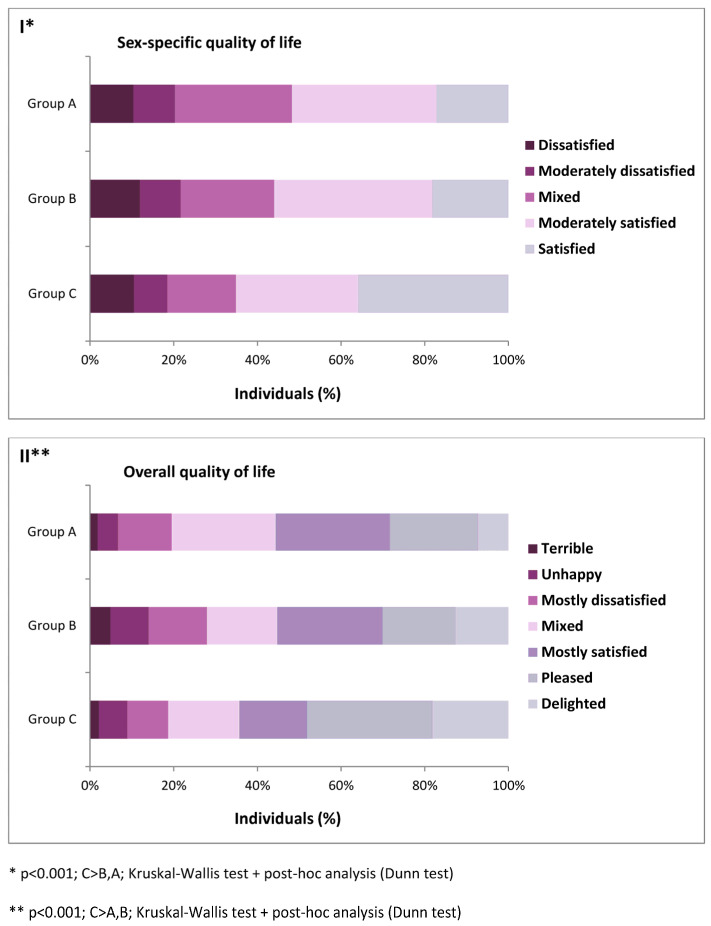
Correlation between lower urinary tract symptoms (LUTS), erectile dysfunction (ED), and premature ejaculation (PE), and sex-specific (I) and overall (II) quality of life (Group A—respondents with LUTS and ED; Group B—respondents with LUTS and PE; and Group C—remaining participants).

We investigated if coexisting LUTS and ED or LUTS and PE had negative effects on the quality of sexual life. Men with LUTS and ED or LUTS and PE had a worse quality of sexual life than the rest of the participants (Figure 1, *p* < 0.001).

For overall quality of life, we observed similar correlations. Men experiencing both LUTS and ED or LUTS and PE exhibited lower overall quality of life in comparison to other participants (Figure 1, *p* < 0.001).

## 4. Discussion

In this analysis, we report high-quality population-level estimates of the effects of LUTS on men’s sexual health. Our data are of high quality because we surveyed a representative pool of men with well-balanced demographics; further, the participants completed reliable questionnaires for evaluation of LUTS, ED, and PE. In addition, our analysis included many covariates, mainly comorbidities and lifestyle habits.

Using regression models, we showed that LUTS significantly affected ED and PE. Essentially, the effects of LUTS on ED and PE remained significant after adjusting for age, comorbidities, and lifestyle factors. In addition, the coexistence of LUTS and ED or LUTS and PE decreased both the quality of sexual life and the general quality of life. However, LUTS did not have a significant effect on men’s sexual activity, as measured by the frequency of sexual intercourse and by the number of sexual partners.

There is a wealth of published data that supports a link between LUTS and ED, including studies that considered potential confounding factors and effect modifiers [24]. Population- and community-based studies that were conducted in different regions of the world, and that were based on different tools to evaluate LUTS and ED, have provided strong evidence of associations between LUTS and ED [25]. Some epidemiological analyses confirmed that the correlations between LUTS and ED were independent of age and comorbidities [6]. However, only a limited number of studies have focused on country-representative pools of respondents. The Multi-National Survey of the Ageing Male-7 (MSAM-7), conducted in the USA and in six European countries (France, Germany, Italy, the Netherlands, Spain, and the UK), is one of the first representative studies that analyzed the association between LUTS and ED. The study included 12,815 men, representative of the population aged 50–80 years in each country [5]. The study showed that ED was strongly related to LUTS and that this relation was independent of reported comorbidities. Shiri et al. surveyed 1716 men representative of the Finnish population and demonstrated that adjusted ORs of ED for men with LUTS were between 2.6 and 4.4 [26]. Other studies that were representative for specific communities but not for entire countries (e.g., Birmingham, UK; Boxmeer, Netherlands; Auxerre, France; Seoul, Korea; Cologne, Germany; and Boston, US) also showed that LUTS is an age-independent risk factor for developing ED [9,10,27]. Therefore, we must recognize that LUTS and ED affect people worldwide. Importantly, the occurrence of LUTS/ED appears mostly independent of environmental and genetic influences because those factors vary substantially between the locations in which LUTS/ED surveys have been conducted. Our study, performed with a representative group of the uniform Polish population, seems to support this hypothesis.

Investigators have assessed correlations between LUTS and PE to a far lesser degree compared with studies of LUTS and ED. A recent systematic review identified twelve studies on associations between LUTS and PE [3]. Although most of the twelve studies showed a significant relation between LUTS and PE, only one study was population-representative. Wein et al. analyzed the impact of LUTS on men’s sexual health in a group of 11,834 representative men who lived in the USA, UK, and Sweden [11]. Their regression models showed that LUTS were significantly associated with PE, but the authors underlined the fact that their PE model was less robust (with a concordance index of 0.62) than the other logistic regression analyses; therefore, the authors recommended caution in interpreting their results. Notably, the researchers assessed PE using solely a self-reported single-item question regarding ejaculation control. In our study with the PEDT, a well-known instrument with high specificity and sensitivity in detecting PE [2], we showed a significant correlation between LUTS and PE, and notably, the effect of LUTS on PE was not influenced by age, comorbidities, or lifestyle factors.

Although we observed the negative effects of coexisting LUTS and ED or LUTS and PE on both quality of sexual life and overall quality of life, our regression models did not validate the effect of LUTS on men’s sexual activity concerning the frequency of sexual intercourse and the number of sexual partners. Our observation might not agree with findings from other studies that showed LUTS as a negative predictor of men’s sexual life. Interestingly, we identified relatively few studies that specifically evaluated the effect of LUTS on men’s sexual activity in terms of frequency of sexual intercourse and number of sex partners. Rosen et al., in the previously cited MSAM-7 study, revealed a significant association between the level of sexual intercourse and the patient’s IPSS [5]. Fwu et al. showed that severe LUTS were cross-sectionally associated with poor sexual drive, which was assessed with the first two questions of the Brief Male Sexual Function Inventory [28]. However, the authors did not observe the same relation longitudinally. Chung et al. similarly demonstrated that LUTS significantly affected sexual drive, but the effect was marginal [29]. Importantly, the effect of LUTS on sexual drive in a non-age-adjusted analysis was lower than the effect of LUTS on erectile functioning, ejaculatory functioning, problem assessment, and overall sexual satisfaction. Further, sexual drive was not correlated within all age strata (correlation coefficients from −0.06 to −0.11; *p* > 0.05), i.e., age-stratified analysis did not provide evidence of an association between LUTS and sexual drive. Therefore, are men with LUTS at greater risk of decreased sexual activity and a poorer sexual life? To answer this important question, we must regard low sexual activity and poor sexual life to be more than just ED or PE, and sexual activity and sexual life should be assessed from several different aspects or ‘domains’ of overall sexual function [2]. We suggest that the association between men’s sexual activity and LUTS is more intricate than initially presumed, and the underlying mechanisms remain incompletely elucidated.

This analysis was not free from limitations. The cross-sectional design limited us to longitudinal assessments of correlations between LUTS, ED, and PE. All data were self-reported; hence, we could not verify any outcome by clinical diagnosis, including lower urinary tract functioning and sexual dysfunctions, a recognized limitation of self-reporting in population-based studies [30].

## 5. Conclusions

In this study, with well-balanced demographic characteristics, we confirmed the contributory effects of LUTS on ED and PE. Notably, the associations between LUTS and ED or LUTS and PE remained unaffected by age, comorbidities, and lifestyle habits. Although respondents with LUTS and ED or LUTS and PE had worse quality of sexual life and general quality of life, we did not observe the effect of LUTS on men’s sexual activity concerning the frequency or the number of partners. Despite this observation, we recommend screening for LUTS in men who report ED or PE.

## Figures and Tables

**Table 1:** The effect of lower urinary tract symptoms (LUTS) on erectile dysfunction (ED) and premature ejaculation (PE).

	LUTS				*p*
	IPSS 0 (A): No Symptoms (*n* = 303)	IPSS 1–7 (B): Mild Symptoms (*n* = 1354)	IPSS 8–19 (C): Moderate Symptoms (*n* = 1068)	IPSS 20–35 (D): Severe Symptoms (*n* = 276)	
ED					
IIEF score: 16 points and less	69 (22.77%)	224 (16.54%)	473 (44.29%)	136 (49.28%)	*p* < 0.001 ^a^
IIEF score: more than 16 points	234 (77.23%)	1130 (83.46%)	595 (55.71%)	140 (50.72%)	
IIEF score: 22–25 ‘without ED’	184 (60.73%)	682 (50.37%)	250 (23.41%)	51 (18.48%)	*p* < 0.001 ^b^ D, C > A, B
IIEF score: 17–21 ‘mild ED’	50 (16.50%)	448 (33.09%)	345 (32.30%)	89 (32.25%)	
IIEF score: 12–16 ‘mild to moderate ED’	24 (7.92%)	149 (11.00%)	336 (31.46%)	97 (35.14%)	
IIEF score: 8–11 ‘moderate ED’	22 (7.26%)	44 (3.25%)	108 (10.11%)	26 (9.42%)	
IIEF score: 5–7 ‘severe ED’	23 (7.59%)	31 (2.29%)	29 (2.72%)	13 (4.71%)	
PE					
PEDT score: 11 points and more	31 (10.23%)	215 (15.88%)	227 (21.25%)	105 (38.04%)	*p* < 0.001 ^a^
PEDT score: less than 11 points	272 (89.77%)	1139 (84.12%)	841 (78.75%)	171 (61.96%)	
PEDT score: 0–8 ‘without PE’	243 (80.20%)	964 (71.20%)	561 (52.53%)	91 (32.97%)	*p* < 0.001 ^b^ D > C > B > A
PEDT score: 9–10 ‘probable presence of PE’	29 (9.57%)	175 (12.92%)	280 (26.22%)	80 (28.99%)	
PEDT score: 11–20 ‘presence of PE’	31 (10.23%)	215 (15.88%)	227 (21.25%)	105 (38.04%)	

^a^ *p*—chi-square test; ^b^ *p*—Kruskal–Wallis test.

**Table 2:** The effect of lower urinary tract symptoms (LUTS) on erectile dysfunction (ED) and premature ejaculation (PE) by age groups (raw scores).

ED				
Age Group	Coefficient	95% CI		*p*
All age groups	−0.224	−0.248	−0.201	<0.001 *
18–24	−0.136	−0.221	−0.052	0.002 *
25–34	−0.208	−0.258	−0.158	<0.001 *
35–44	−0.258	−0.302	−0.214	<0.001 *
45–54	−0.132	−0.189	−0.074	<0.001 *
55–64	−0.224	−0.274	−0.175	<0.001 *
65 and more	−0.255	−0.333	−0.176	<0.001 *
	**PE**			
**Age Group**	**Coefficient**	**95% CI**		***p***
All age groups	0.592	0.535	0.65	<0.001 *
18–24	0.43	0.241	0.619	<0.001 *
25–34	0.744	0.622	0.866	<0.001 *
35–44	0.505	0.392	0.617	<0.001 *
45–54	0.672	0.548	0.796	<0.001 *
55–64	0.553	0.414	0.692	<0.001 *
65 and more	0.65	0.423	0.877	<0.001 *

*p*—univariate linear regression; * statistical significance (*p* < 0.05).

**Table 3:** The effect of lower urinary tract symptoms (LUTS) on erectile dysfunction (ED) and premature ejaculation (PE)—multivariable linear regression.

		ED				PE			
Variable		Parameter	95% CI		*p*	Parameter	95% CI		*p*
LUTS		−0.159	−0.184	−0.135	<0.001 *	0.528	0.474	0.583	<0.001 *
Age	18–24	ref.				ref.			
	25–34	0.785	0.153	1.417	0.015 *	−0.628	−1.51	0.253	0.162
	35–44	1.359	0.743	1.974	<0.001 *	−0.740	−1.633	0.216	0.14
	45–54	0.962	0.313	1.612	0.004 *	−0.846	−1.25	0.641	0.41
	55–64	0.673	0.021	1.325	0.043 *	−0.548	−1.456	0.361	0.237
	65 and more	−1.463	−2.255	−0.671	<0.001 *	0.927	−0.177	2.031	0.1
Diabetes		−0.375	−0.984	0.233	0.227	−0.278	−1.126	0.631	0.323
Depression		−1.188	−1.853	−0.523	<0.001 *	1.541	0.616	2.466	0.001 *
Any pulmonary disease		−0.449	−1.137	0.239	0.201	0.755	−0.499	1.711	0.125
Any cardiac disease		−0.476	−1.179	0.228	0.185	0.71	−0.27	1.69	0.156
Arterial hypertension		0.145	−0.318	0.609	0.539	−0.044	−0.691	0.602	0.893
Lipid disorders		−0.722	−1.216	−0.227	0.004 *	0.238	−0.451	0.928	0.498
Myocardial infarction		−0.332	−1.141	0.478	0.422	0.399	−0.73	1.529	0.488
Stroke		−0.05	−0.917	0.817	0.91	0.881	−0.474	2.088	0.239
Smoking		0.23	−0.122	0.582	0.201	0.174	−0.317	0.664	0.488
Overweight/obesity		−0.273	−0.643	0.098	0.149	−0.617	−0.132	0.901	0.197
Excessive alcohol intake ^1^		−0.244	−0.715	0.227	0.31	1.215	−0.559	0.87	0.432
Polypragmasy ^2^		−1.087	−1.644	−0.53	<0.001 *	1.468	−0.693	1.242	0.285
Any surgeries in abdomen or pelvis		0.122	−0.401	0.644	0.648	0.128	−0.6	0.857	0.73

*p*—multivariable linear regression; * statistical significance (*p* < 0.05); ^1^ excessive alcohol intake was defined as two or more alcohol portions per day; ^2^ polypragmasy was defined as five or more medications per day.

**Table 4:** The effect of IPSS questions on the IIEF and PEDT scores.

		IIEF Score				PEDT Score		
Variable	Coefficient	95% CI		*p*	Coefficient	95% CI		*p*
IPSS item 1	−0.425	−0.608	−0.243	<0.001 *	0.431	0.278	0.584	<0.001 *
IPSS item 2	0.112	−0.135	0.288	0.001	0.178	0.03	0.327	0.019 *
IPSS item 3	−0.307	−0.502	−0.112	0.002 *	−0.118	−0.281	0.046	0.159
IPSS item 4	−0.362	−0.546	−0.177	<0.001 *	0.387	0.232	0.543	<0.001 *
IPSS item 5	−0.241	−0.445	−0.036	0.021 *	0.405	0.233	0.577	<0.001 *
IPSS item 6	−0.371	−0.56	−0.182	<0.001 *	0.037	−0.122	0.196	0.649
IPSS item 7	−0.124	−0.271	0.022	0.097	0.094	−0.03	0.217	0.137

*p*—multivariate linear regression; * statistical significance (*p* < 0.05).

## Data Availability

All data generated or analyzed during this study are included in this published article.
